# Challenges towards quality assurance of Basic Medical Education in Pakistan

**DOI:** 10.12669/pjms.36.2.1319

**Published:** 2020

**Authors:** Abdul Waheed Khan, Ahsan Sethi, Gohar Wajid, Raheela Yasmeen

**Affiliations:** 1 Dr. Abdul Waheed Khan, MBBS, MSC, MPH, MHPE. Department of Medical Education, Riphah International University, Islamabad, Pakistan; 2 Dr. Ahsan Sethi, BDS, MPH, MMEd, FHEA, MAcadMEd, PhD Medical Education, Assistant Professor, Institute of Health Professions Education and Research, Khyber Medical University, Pakistan; 3 Dr. Gohar Wajid, MBBS, MSc, MPH, PhD Medical Education. Consultant, Health Professions Education, Department of Medical Education, Riphah International University, Islamabad, Pakistan; 4 Prof. Raheela Yasmeen, BDS, DCPS-HPE, JMHPE, MHPE. Department of Medical Education, Riphah International University, Islamabad, Pakistan

**Keywords:** Challenges, Basic Medical Education, Accreditation, Quality Assurance

## Abstract

**Objective:**

There are growing concerns towards the quality of medical education in Pakistan. To help strengthen accreditation processes, this study identifies the challenges towards quality assurance of Basic Medical Education in Pakistan.

**Methods:**

A qualitative case study was carried out from March to August 2018. Participants included inspectors from various disciplines in both public and private medical colleges, and medical educationists from Pakistan. Semi-structured interviews were conducted with 12 inspectors, while focus group discussion included 10 medical educationists. All the interviews were audio recorded and transcribed verbatim. Thematic analysis was conducted to capture the intricacies of meaning within the data.

**Results:**

Data identified 14 sub-themes grouped under three major themes. Challenges towards quality assurance included mounting political influence, commercialism in medical education, weak regulatory capacity of accrediting body, violation of rules, lack of valid accreditation standards and skilled inspectors.

**Conclusion:**

Quality assurance of Basic Medical Education in Pakistan involves various systemic, resource and personnel related challenges. The accrediting body needs to bring major reforms in its accreditation system and strengthen its regulatory and technical educational capacity to ensure the quality of medical education in nearly 168 medical and dental colleges of the country.

## INTRODUCTION

In response to the increasing health workforce needs of Pakistan, the number of medical and dental colleges have increased from 22 in 1990 to 168 in 2019.^1^ With this rapid increase, there are continuing concerns about the quality of education in these colleges.^2^ The shortage of trained faculty, especially in basic medical sciences, remains a major challenge.^3^ Irrespective of the fact that how many doctors are produced and deployed, health professionals cannot contribute to population health and wellbeing effectively, unless they acquire essential competencies through high quality medical education.^4^

Pakistan Medical and Dental Council (PM&DC) is the sole authority for accrediting and regulating undergraduate medical/dental education in Pakistan. It helps ensure that the quality of medical education is in line with the evolving needs of the healthcare delivery system and expectations of society.^5^ Medical educators are showing increasing concerns about the nature of current PM&DC accreditation standards, the accreditation processes and the limited technical capacity of the regulatory body to ensure the quality of medical education in the country.^5^

First set of PM&DC regulations were developed in 1962 and the most recent revisions were published in 2019.^6^ The existing inspection proformas are mainly input based (emphasizing the presence of infrastructure in medical colleges), without taking the educational processes and quality of outcomes into much consideration.^7^ Until recently, these regulations were taken implicitly as the ‘PM&DC standards’, implemented through an ad-hoc based team of inspectors, exercising their authority through performing ‘inspection’ of the medical colleges. Quality was assumed to be assured if the team declares the college as meeting minimum criteria.

In response to the national^5^ and international^8^ calls for strengthening accreditation system and standards, PM&DC recently developed new standards.^9^ Although influenced by World Federation for Medical Education (WFME) standards,^10^,^11^ the PM&DC standards are contextualized to medical education system in Pakistan. Though the ‘inspection’ function has been renamed as ‘accreditation’, the transformation of the function from ‘inspection to accreditation’ has not been fully achieved yet. The new standards are being tested for their validity, measurability, acceptability and compatibility with both the local context and changing global scenario. Gaps also exist in improving PM&DC technical capacity to perform accreditation and provide training to quality assurance units in medical colleges to prepare colleges to meet new standards. To help strengthen PM&DC accreditation processes, this study identifies the challenges towards quality assurance of Basic Medical Education in Pakistan.

## METHODS

This qualitative case study was carried out over six months (March-August 2018). Ethical Review Committee, (Riphah/IIMC/ERC/18/0236, Dated: February 6, 2018) Riphah International University granted approval for the study. Sampling frame included diverse range of stakeholders i.e. faculty from various disciplines in both public and private medical colleges, accreditation body staff and medical educationists involved in quality assurance of Basic Medical Education in Pakistan.

### Interview Guide

An interview guide was developed, piloted and revised after through deliberation. Questions were asked about the current accreditation system for recognition of medical and dental colleges. The capacity of accrediting body to facilitate the accreditation processes, its strengths and weaknesses. Strategies adopted by the medical colleges to prepare themselves to comply with the accreditation requirements and issues and challenges inspectors face during the accreditation process.

### Data Collection

Participants were selected through Purposive, Maximum Variation Sampling. Voluntary nature of participation was explained to all participants and an informed consent was taken. Individual interviews were conducted with 12 inspectors for focused two-way communication that encouraged rich descriptions of participants’ experiences. A focus group discussion was also conducted with 10 medical educationists attending International Conference on Health Professions Education and Research 2018 at Khyber Medical University, Pakistan. The sample size was not predetermined, and an iterative approach of simultaneous data collection and analysis was taken until saturation was achieved. All the interviews were audio recorded and transcribed verbatim.

### Data Analysis

Thematic analysis was conducted to capture the intricacies of meaning within the data. Each line and segment in the data was carefully read to construct in-vivo analytic codes for fitness and relevance. The selective codes and associated data were categorized, and themes were developed.^12^ The themes were refined continuously through reflective thinking, memo writing and team discussions.

## RESULTS

Individual interviewees (n=12) and focus group discussion participants (n=10) were at varying stages in their professional careers and from different provinces of Pakistan. Their professional background was predominantly medicine and included: a vice chancellor, deans, principals of colleges, faculty from various specialties and directors. Seven focus group participants were renowned medical educationists of the country (Table-I).

**Table-I:** Participant Characteristics.

Characteristics		Individual Interviewees (n=12)	Focus Group Participants (n=10)
Gender	Male	11	9
	Female	1	1
Age	<40 Yrs.	1	1
	40-49 Yrs.	2	3
	50-59 Yrs.	3	5
	>59 Yrs.	6	1
Workplace	Punjab	9	3
	Khyber Pakhtunkhwa	2	3
	Sindh	1	4
Qualifications	PhD	2	4
	Fellowship/MPhil	8	3
	Masters	2	3
Faculty Type	Clinical Sciences	8	2
	Basic Sciences	3	
	Dentistry	1	1
	Medical Education		7
Rank	Vice Chancellor		1
	Dean/Principal	3	2
	Professor	7	2
	Member of the accrediting body	1	
	Staff of the accrediting body	1	
	Directors		2
	Assistant Professor		3
Inspection Experience	Inspectors	10	6
	Member of the accrediting body	1	1
	Co-opt committee member of the accrediting body		3
	Coordinator Inspection Team	1	

Through analysis of the transcripts, we identified 14 sub-themes grouped under three major themes (Table-II). These challenges are not mutually exclusive and do not occur in isolation.

**Table-II:** Challenges towards Quality Assurance of Basic Medical Education.

Inhibiting Systemic Factors	Mounting political influence	With constitution of the new council, every member tried to work on merit, but political pressure did not allow to move the proceedings smoothly. That is why (accrediting body) lost its credibility and the public confidence.
	Bias in the selection of inspectors	Selection criteria lacks objectivity. There is a long list of inspectors but (accrediting body) select them by their wish…it is all random…like the berry picking…there is no uniform policy.
	Lack of valid accreditation standards	The accreditation process laid down by the (accrediting body) does not focus on quality. It focuses simply on quantity. It is very important to actually know what these students are learning not just where they are learning.
	Public-Private sector divide	The inspectors are…more lenient to the public-sector medical colleges and are harsh to the private-sector medical colleges. The standard is not uniform.
	Rapid growth of medical colleges	Exponential increase in the number of medical colleges, has led to the deficiency of skilled faculty especially in the basic science subjects.
	Increased faculty turnover	Faculty members…do not own their students and they cash their qualification in terms of money…they enter in one medical college and after a year they go to other medical college with attractive salary.
	Commercialism in medical education	The element of commercialization has played a havoc with medical education. Proper education and training is not given, especially in private medical colleges. Medical education has gone to one side and commercial element has become more pronounced.
	Unethical practices of medical colleges for accreditation	The college owners were willing and offering financial gains…for the recognition of their substandard medical colleges…During a college inspection, we found that there were some laborers working in the building of that medical college…later on we found those laborers lying on the [hospital] beds.
Inhibiting Resource Factors	Ad hoc members and committees	President/vice president of (accrediting body) are non-residents. You know calling a session after a month and coming from different places…leaving behind institution as well as private practice, so they don’t work with full concentration and selflessness which is desired by such a notable organization.
	Accrediting body technical capacity	The technical expertise at the secretariat is also deficient. We are still doing manual work. IT reforms are needed badly along with human resource, which means that not only the number, but the technical as well as professional experts in relevant disciplines must be increased.
	Inspection time	Seven to 8 hours is the period in which all departments have to be inspected and then you have to return…this time is insufficient for thorough assessment.
Inhibiting Personnel Factors	Incompetent a.Members b.Inspectors	There were certain in-house fights among members, because immature and inexperienced… doctors came into (accrediting body). Inspectors are not trained…they face problems and…miss important component of inspection.

*Anonymized the organization (accrediting body).

### Inhibiting Systemic Factors

Among systemic factors, the participants believed that the biggest challenge is the use of negative political influence, both by the government and private college owners to get their colleges accredited despite having major gaps in standards compliance. Most appointments at the regulatory authority including that of its president and members were politically influenced, which then effected decisions pertaining to recognition of medical colleges. Participants also reported the lack of valid accreditation standards as a challenge. They mentioned that current accreditation system primarily focuses on infrastructure evaluation only. The colleges should also be evaluated on the quality of education they impart, the quality of their curriculum and assessment processes. The participants also reported an inherent bias in the evaluation of public-sector institutions in comparison to private-sector institutions. Other challenges included the mushroom growth of medical colleges with insufficient faculty. High faculty turnover was also mentioned as a major challenge. The increased commercialism in medical education also resulted in medical colleges being seen as a money-making business opportunity with no social or self-accountability. Weak accreditation process renders accreditation as one-time activity, repeated every five years. Medical colleges may adopt a number of unethical and even illegal ad-hoc practices to get through the process of accreditation. Once the accreditation target is achieved, they fall back to their original sub-standard educational practices.

### Inhibiting Resource Factors

The participants referred to the ad hoc nature of members and lack of technical capacity within the accrediting body as major challenges. In most cases, the time for inspection is also insufficient for a rigorous and thorough inspection process.

### Inhibiting Personnel Factors

At personnel level, the key challenges included the incompetence of the accrediting body members and inspectors. The members of the accrediting body did not understand the accreditation process and the role of accrediting body. They were incapable of handling the responsibilities due to their lack of knowledge and experience. Moreover, the inspectors involved in the accreditation process were not formally trained, affecting the reliability and validity of their evaluation of the institutes and programmes.

## DISCUSSION

The study highlights the challenges towards quality of Basic Medical Education in Pakistan. Our findings are in line with challenges reported by World Health Organization i.e., lack of strong medical education regulatory system and quality assurance processes.^4^ A robust accreditation system for medical institutions is required for the credibility of medical teaching programs, and performance of medical colleges in imparting high quality teaching and training to the medical students,^13^ which in turn ensures competent doctors and patient safety.^14^

The current study highlighted this politicization of the accrediting body, as a major challenge, affecting the quality of medical education in Pakistan. For many years, the members of accrediting body in Pakistan included principals of the colleges, nominees of the universities syndicate, Surgeon General (armed forces), elected representatives of general practitioners, Director General of Health and federal/provincial secretaries. With mushroom growth of private-sector colleges, the number of council members exceeded 100, making the functioning of the accrediting body difficult. In 2012, the number of members was reduced through an ordinance and included nominees of national assembly/senate, federal/provincial government and elected representatives from public/private sector. This resulted in negative political influence on most appointments and did not improve the functioning of the regulatory body. In 2015 and then in 2018, further changes were introduced in the structure of the accrediting body, however, these have not been approved by the national assembly/senate as yet.

The concept of “political spectacle” introduced by Edelman and colleagues, explains that politics garble the educational policies. Political spectacle opposes equalitarian, compensatory and communitarian values. Instead political spectacle addresses special interest of few, often political giants, that hide behind common good, as the distribution of goods is veiled backstage.^15^ The ill-logical political decisions ostracize the role and contribution of professionals with negative impact on educational policy formulation and implementation. Studies confirm a negative impact of politicization on the performance of public agencies, which is partially mediated by politicization’s deleterious effect on their human resource management.^16^ Therefore, we recommend that the accrediting body should comprise of members nominated/selected based on their qualification, experience and professional standing by an independent board and not on political basis.

Our study revealed some inherent weaknesses and flaws in the accreditation regulations, which hampers autonomy of the accrediting body that directly or indirectly affects the accreditation process. As per literature, many regulatory authorities are formally shielded from direct political influence and thus enjoy high level of legal autonomy. In the United States, the educational accrediting agency is a powerful instrumentality with minimal governmental interference, to set policies and standards in an area of vital concern to the public.^17^ Credible accreditation agencies, spell a clear eligibility criteria for the selection of inspectors, and pay special attention for their training.^18^ Whereas current study revealed that our selection and decisions-making processes are vague and non-standardized.

Another challenge to our educational quality assurance is heavy emphasis on ‘structure based’ standards, primarily focusing on physical infrastructure and material resources.^5^ As per literature, commonly used accreditation model for medical education is ‘the process model’.^19^ It comprises of self-evaluation on the basis of recognized standards, followed by a site visit by trained inspectors and a report highlighting the outcome of the inspection.^19^ The accrediting body must establish standards, or follow model standards for assessing the delivery of medical education and training.^20^ WFME standards for Basic Medical Education are mostly qualitative and process oriented and increasingly being adapted and adopted by the accrediting bodies globally.^21^ Australian Medical Council has also developed new set of standards for accreditation.^22^

Commercialization and mushrooming of medical colleges were also highlighted as challenges. While the role of private sector in education is inevitable, the government and the regulatory body must ensure that clear policies are in place to open new medical colleges and ensure that the quality of medical education is not compromised. Educational standards must be applicable to both public and private sector colleges in a uniform manner to ensure uniformity in the quality of medical education.^23^

### Limitations of the study

Most study participants belonged to the medical colleges of Punjab and Khyber Pakhtunkhwa with fewer participants from Sindh and Baluchistan provinces. Despite the limitations, the findings offer an understanding of the challenges and identified areas for improvement.

## CONCLUSION

There are numerous challenges to the quality of Basic Medical Education in Pakistan. The foremost and important challenges include the mounting political influence, commercialism in medical education, weak regulatory capacity of accrediting body, violation of rules, lack of skilled inspectors and objective assessment criteria. The regulatory body should review its accreditation system and strengthen its regulatory and technical educational capacity to ensure the quality of medical education in nearly 168 medical and dental colleges of the country.

### Authors’ Contributions:

**AWK, GW** and **AS** conceived the idea and designed the study, are responsible for integrity of research.

**AWK** and **GW** were involved in data collection.

**AWK, AS, GW** and **RY** did the data analysis and data interpretation.

All the authors contributed towards writing the manuscript, approved the final version.
